# Effects of Texas State Agency Integration on Mental Health Service Use Among Individuals with Co-occurring Cognitive Disabilities and Mental Health Conditions

**DOI:** 10.1007/s10597-024-01332-0

**Published:** 2024-08-01

**Authors:** Elizabeth M. Stone, Andrew D. Jopson, Nicholas J. Seewald, Elizabeth A. Stuart, Elizabeth Wise, Alexander D. McCourt, Danielle German, Emma E. McGinty

**Affiliations:** 1 Department of Psychiatry, Rutgers Robert Wood Johnson Medical School, New Brunswick, NJ USA; 2 Department of Health Policy and Management, Johns Hopkins Bloomberg School of Public Health, Baltimore, MD USA; 3 Department of Biostatistics, Epidemiology, and Informatics, Perelman School of Medicine, University of Pennsylvania, Philadelphia, PA USA; 4 Department of Biostatistics, Johns Hopkins Bloomberg School of Public Health, Baltimore, MD USA; 5 Department of Psychiatry and Behavioral Sciences, Johns Hopkins School of Medicine, Baltimore, MD USA; 6 Department of Health, Behavior and Society, Johns Hopkins Bloomberg School of Public Health, Baltimore, MD USA; 7 Division of Health Policy and Economics, Department of Population Health Sciences, Weill Cornell Medicine, New York, NY USA; 8 Center for Health Services Research, Rutgers Institute for Health, Health Care Policy, and Aging Research, New Brunswick, NJ USA

**Keywords:** Cognitive disability, Intellectual disability, Developmental disability, Mental health, State government, Administrative agencies

## Abstract

This study uses Texas’s 2017 integration of the state disability and mental health agencies as a case study, combining interviews with Texas agency and advocacy organization leaders to examine perceptions of agency integration and augmented synthetic control analyses of 2014–2020 Medical Expenditure Panel Survey to examine impacts on mental health service use among individuals with co-occurring cognitive disabilities (including intellectual and developmental disabilities) and mental health conditions. Interviewees described the intensive process of agency integration and identified primarily positive (e.g., decreased administrative burden) impacts of integration. Quantitative analyses indicated no effects of integration on receipt of mental health-related services among people with co-occurring conditions. While leaders identified some potentially beneficial impacts of state agency integration, the limited impact of integration beyond the agency suggests that interventions at multiple levels of the service system, including those targeting providers, are needed to better meet the mental health service needs for this population.

**Supplementary Information:**

The online version contains supplementary material available at 10.1007/s10597-024-01332-0.

## Introduction

People with intellectual and developmental disabilities (IDD) such as Down syndrome or autism spectrum disorder have high prevalence of mental health conditions including depression and anxiety. More than 1 in 3 individuals with IDD have a mental health condition (Mazza et al., 2020) compared to 1 in 4 individuals in the general population (SAMHSA, 2022). Despite the elevated prevalence of mental health conditions among individuals with IDD, this population faces significant barriers to receiving mental health care (Durbin et al., 2017; Krahn et al., 2006; Whittle et al., 2018). One key barrier is fragmentation of the mental health and IDD service systems (Durbin et al., 2017; Ervin et al., 2014; Krahn et al., 2006; Kramer et al., 2019; NASMHPD, 2017; Peña-Salazar et al., 2020; Pinals et al., 2021a, 2021b; Whittle et al., 2018). Fragmentation of services frequently results in individuals with co-occurring IDD and mental health conditions receiving care from providers who are unfamiliar with their needs (Ervin et al., 2014; Kramer et al., 2019; Pinals et al., 2021a; Whittle et al., 2018). These silos also increase the administrative burden on individuals seeking treatment, requiring people to navigate multiple providers, eligibility requirements, and payment systems (Ervin et al., 2014; NASMHPD, 2017; Pinals et al., 2021b). System fragmentation ultimately results in significant unmet mental health care needs for individuals with co-occurring IDD and mental health conditions (Kramer et al., 2019; Peña-Salazar et al., 2020).

One critical area where fragmentation could be addressed is in strengthening connections between the state agencies that oversee and regulate services for people with co-occurring IDD and mental health conditions. Of the 51 state IDD agencies (including Washington DC), 39 are offices or divisions within larger state executive agencies (e.g., the state department of health), six are divisions within mental health agencies, and six are stand-alone IDD-specific agencies (NASDDDS, 2022). State agencies have important roles in allocating funding, regulating service sectors, monitoring and evaluating performance and outcomes, and providing services directly (NASMHPD, 2012). When individuals with both IDD and mental health conditions are required to access services overseen by separate agencies, there may be differing structures, eligibility rules, clinical programs, and financing mechanisms that impede coordination of care (NASMHPD, 2017; Pinals et al., 2021b; Whittle et al., 2018). By strengthening connections between IDD and mental health agencies, states can facilitate formal and informal cross-sector partnerships, establish consistent regulatory and eligibility requirements across service sectors, promote training and provide technical assistance for providers working at this intersection, and pool resources to more efficiently provide services (Gans & Horton, 1975; SAMHSA, 2021). These connections have the possibility to improve access to and quality of services for people with IDD and mental health conditions.

Texas’s integration of the Department of Aging and Disability Services (DADS) and the mental health agency provides a unique natural experiment to evaluate the effects of agency integration on mental health services for people with co-occurring cognitive disabilities including IDD. Following recommendations of a Sunset Advisory Commission, the 2015 Texas legislature passed a bill calling for a transformation of the state Health and Human Services Commission effective September 2017 (Texas HHS, 2018; n.d.). This included the elimination of DADS, the state agency responsible for individuals with IDD, other disabilities, and aging-related needs, and the integration of its programmatic and regulatory responsibilities into the department of mental health within the Health and Human Services Commission. The stated purpose of this integration was to “make it easier for people to find out about services or benefits for which they might qualify; better integrate similar programs and services together, removing bureaucratic silos; create clear lines of accountability within the organization; and include well-defined and objective performance metrics for all organizational areas” (Texas HHS, n.d.).

The present analysis uses a concurrent triangulation mixed methods design to (1) examine the process and perceptions of agency integration from those involved, focusing on impacts related to individuals with co-occurring IDD and mental health conditions specifically and (2) assess the effects of integration on mental health service use among individuals with co-occurring cognitive disabilities, such as IDD, and mental health conditions. This approach allows us to leverage the relative strengths of both qualitative and quantitative data methods and to examine complementary information, providing a more holistic overview of the integration process. We hypothesize that integrating state mental health and disability agencies may improve individual mental health service outcomes (i.e., decrease inpatient and emergency department service use and increase outpatient service use related to mental health conditions) by combining agency resources and leveraging both mental health and IDD expertise in the regulation, provision, and training related to mental health services for individuals with both IDD and mental health conditions.

## Methods

This study used a concurrent triangulation mixed methods design intended to provide a more holistic view of agency integration by employing complementary qualitative and quantitative data methods (Creswell & Clark, 2017). We simultaneously collected and analyzed qualitative and quantitative data. Following separate analysis, qualitative and quantitative results were considered together for interpretation and development of conclusions related to the effects of the agency integration in Texas on mental health services for adults with IDD and mental health conditions. This study was reviewed and approved by the Johns Hopkins Bloomberg School of Public Health Institutional Review Board.

### Qualitative Methods

#### Sample, Recruitment, and Instrument

We conducted semi-structured interviews via the Zoom videoconferencing platform with state agency and advocacy organization leaders in Texas with expertise in mental health services for individuals with IDD and mental health conditions living in the state. Potential interviewees were identified through a combination of purposive and snowball sampling. We initially contacted state agency staff in the IDD-Behavioral Health Services Department at the Texas Health and Human Services Commission and directors of advocacy organizations representing individuals with IDD and/or mental health conditions and service providers for these populations. Identified individuals were asked to participate in the study and to recommend others who may have relevant knowledge. All potential interviewees were contacted by an initial email that explained the study’s purpose and goals. Two follow-up emails, each one week apart, were sent in the case of non-response. Interviews continued until stakeholders did not have additional suggestions for potential interviewees. A common interview guide was used for all interviews (Appendix A in Supplementary material). Interview domains related to state agency integration were informed by the “Process” construct from the Consolidated Framework for Implementation Research (CFIR) and questions focused on the role of state agencies in mental health services for people with both IDD and mental health conditions, the process of agency integration (CFIR constructs planning, engaging, and executing), and the perceived outcomes of integration (CFIR construct reflecting and evaluating) (Damschroder et al., 2009).

Interviews were conducted from April to July 2022 by one study team member with prior training and experience in conducting qualitative interviews with state agency leaders. Interviews were digitally recorded and transcribed; verbal consent was obtained at the beginning of each interview, prior to recording. All transcripts were reviewed and validated against the audio recording and identifying information was removed prior to analysis. Interviews ranged from 21 to 70 min in length (median: 31 min).

#### Qualitative Analysis

After completion of each interview, a summary memo was drafted to inform subsequent interviews and codebook development. After all interviews were completed, transcripts were analyzed using a hybrid inductive/deductive coding approach to identify key themes and sub-themes (Guest et al., 2011; Kiger & Varpio, 2020). An initial codebook was developed based on the summary memos and CFIR (Damschroder et al., 2009). Two study team members pilot tested the codebook, independently coding a randomly selected subset of four interviews, to assess differences in coding decisions and identify additional codes that should be included. The final codebook was used by both team members to code all transcripts. Throughout both pilot and final coding, study team members held regular meetings to discuss questions related to the coding process. Codes were then arranged into themes and sub-themes and representative quotes were identified. All interviewees were asked to review preliminary themes and provide feedback to ensure results reflected interviewees’ experience of agency integration. Transcripts were coded using NVivo (QSR International Pty Ltd, 2020).

### Quantitative Methods

#### Data and Study Design

We used Medical Expenditure Panel Survey (MEPS) Household Component data from 2014 to 2020. Conducted by the Agency for Healthcare Research and Quality (AHRQ), MEPS is a large, nationally representative survey of civilian, non-institutionalized families and individuals (AHRQ, 2019). In the panel design, sampled households are surveyed in multiple rounds (5 or 7) over a 2 year period (AHRQ, 2019). This survey data is supplemented by information provided by individuals’ employers and medical providers including hospitals, physicians, home health care providers, and pharmacies, providing a unique combination of survey and healthcare utilization data (AHRQ, 2019).

In this analysis, Texas was the sole treatment state. Control states were selected by identifying states in which the IDD and mental health agencies were separate (i.e., not under the same umbrella agency) for the entire study period. Eleven states met this requirement (AZ, CT, FL, NM, NY, OH, OK, OR, SC, SD, TN). State-specific MEPS data was not available for South Dakota due to small sample size, so this state was excluded from final analysis.

#### Study Population

We subset the MEPS data to 1644 total individuals (347 in Texas and 1297 in the ten control states) with cognitive disabilities and mental health conditions during the study period (2014–2020). For this analysis, we included both children and adults. Because MEPS does not include a specific measure of IDD, we follow prior research in creating a sample of individuals with cognitive limitations which would include individuals with IDD in addition to people with cognitive limitations related to other conditions (e.g., dementia) (Reichard & Stolzle, 2011). Individuals were identified as having a cognitive limitation if they “(1) experienced confusion or memory loss, (2) had problems making decisions, or (3) required supervision for their own safety” (AHRQ, 2019; Reichard & Stolzle, 2011). Individuals were defined as having a mental health condition if they had a relevant psychiatric diagnosis (ICD-9 codes 295–298, 300–302, 306–309, 311–314 or ICD-10 codes F20-F25, F28-F34, F39-F45, F48, F50-F52, F59, F60, F63-F66, F68, F90, F91, F93-F95, F98, F99, G44, R37, R45, Z87) in the medical conditions file (NCQA, 2016). Medical conditions in MEPS are identified by either an individual reporting they have been diagnosed with a certain condition or the condition being identified as the reason for a medical event (e.g., hospital stay or outpatient visit) (AHRQ, 2019). Individuals were considered as having co-occurring cognitive disability and mental health condition if they met both criteria during the same year. Alternate definitions of cognitive disability and mental health conditions were tested in sensitivity analyses. We defined an alternative measure of cognitive disability as either cognitive limitation as described above or serious cognitive difficulty defined as “difficulty concentrating, remembering, or making decisions” (AHRQ, 2019). An alternative measure of a mental health condition was defined as having one of the listed diagnostic codes or a patient health questionnaire (PHQ) score greater than 2 (indicating possible depression) or a Kessler psychological distress scale (K6) score greater than 12 (Kessler et al., 2002; Kroenke et al., 2001).

#### Measures

State agency integration was coded as a binary indicator that changed from 0 to 1 in the implementation year for the treated state (2017 in Texas). Outcome measures included four types of mental health service utilization: inpatient stays, emergency department visits, outpatient visits, and prescription medications for psychiatric illness. Inpatient stays, emergency department visits, and outpatient visits were considered mental health-related if they were associated with a relevant diagnosis code as defined above. Prescription medications were considered mental health-related based on therapeutic class (psychotherapeutic medications). Outcomes were coded as binary indicators of any use of services in a year and as a count of number of services per year among those with at least one service in a given year. For all analyses, data was aggregated to the state-year level.

Covariates constructed using the MEPS sample of individuals with both cognitive disability and mental health conditions included state-year level aggregated measures of age, sex, race/ethnicity, household annual income, highest educational degree attained, employment status, marital status, insurance coverage, autism spectrum disorder diagnosis (ICD-9 code 299 or ICD-10 code F84) mood disorder diagnosis (ICD-9 code 296 or ICD-10 codes F30, F31), anxiety disorder diagnosis (ICD-9 codes 300,309 or ICD-10 codes F40-F45, F48), and schizophrenia diagnosis (ICD-9 code 295 or ICD-10 codes F20, F25) (Lin et al., 2013; NCQA, 2016). For example, demographic information included mean age and the proportion of individuals who were female within each state for each study year.

#### Statistical Analysis

We used an augmented synthetic control approach to examine the impact of state agency integration on mental health service utilization among people with co-occurring cognitive disability and mental health conditions in Texas (Ben-Michael et al., 2021). This method is similar conceptually to a difference-in-differences analysis in that it compares changes in outcomes before and after an intervention in a treated group to changes in outcomes over the same time period in a control group. In this approach, the control group—the “synthetic control”—is the weighted combination of comparison states that best approximates both the trend and magnitude of the outcome in the treated state prior to agency integration. The weighted combination is then carried forward into the post-integration period as an estimate of what would have happened in the treated state in the absence of integration. The augmented synthetic control method adds a parametric outcome model to the standard synthetic control approach to improve the pre-treatment fit (Ben-Michael et al., 2021).

To generate the synthetic control, we used baseline (2014–2016) outcomes and the covariates described above to construct the weighted combination of control states that best approximated the pre-treatment trends in Texas. The synthetic control weights were then augmented with a linear regression model for each outcome to estimate the average treatment effect in the treated state (ATT) (Ben-Michael et al., 2021). The regression models included all covariates listed above and state fixed effects. All analyses were conducted at the state-year level. We report an estimated ATT averaged over the entire post-treatment period and changes in outcomes attributable to agency integration for each post-integration year individually. We include six sensitivity analyses: three with different sample population definitions (alternate definition of cognitive disability (defined above), alternate definition of mental health condition (defined above), and exclusion of individuals aged 55 and older who may be more likely to be identified as having a cognitive limitation due to dementia or other age-related conditions rather than IDD), one using an implementation date of 2016 (as opposed to 2017) which aligns with phase one of agency integration (described below), and two including different sets of covariates in the augmenting outcome model (state fixed effects only and state fixed effects, measures of age, race/ethnicity, insurance coverage, and autism spectrum disorder or mental health diagnosis only). All analyses were conducted using the ‘augsynth’ package in R (Ben-Michael, 2021).

## Results

### Qualitative Results

We conducted interviews with eight Texas stakeholders (three government agency leaders and five advocacy organization leaders) with professional experience related to mental health services for individuals with co-occurring IDD and mental health conditions and knowledge of the agency integration. Interviewees had a mean of 16 years of experience working with individuals with IDD and mental health conditions and had been in their current position for a mean of 5 years. Interviewees described a four-step process of agency integration and expressed both positive and negative impacts of this integration on mental health services for individuals with both IDD and mental health conditions.

**Fig. 1:**
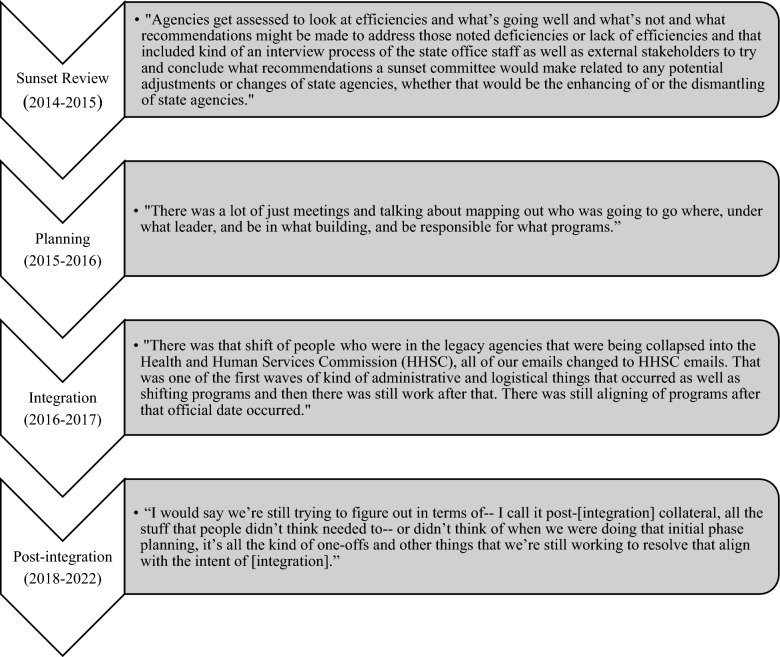
Process of Texas agency integration^a^

The first step of the agency integration process was a Sunset Advisory review (Fig. 1). In Texas, agencies are regularly assessed by the Sunset Advisory Commission in a process that includes both internal (from the agency) and external (from the public) feedback on agency performance. This commission then makes recommendations for legislative action based on the review. In 2015, following review of the Department of Aging and Disability Services, the state IDD agency, the Sunset Advisory Commission recommended consolidation of that agency into the Health and Human Services Commission. This recommendation was then taken up by the Texas legislature. In planning for the integration, workgroups comprised of agency leaders were charged with organizing the agency integration process, making decisions about where to move people and programs and the integration timeline. Agency integration took part in a two-step process. In phase one (September 1, 2016), all client services and programs were moved from the Department of Aging and Disability Services to the Health and Human Services Commission. In phase two (September 1, 2017) facilities and regulatory service programs (e.g., the consumer rights unit) were transferred and the Department of Aging and Disability Services was officially abolished. Interviewees described this as a long and complex process. People and programs were moving physical location in addition to changing organizational affiliation. While there were target deadlines for each phase of the integration, some logistics continued to be worked out even after those dates.

One perceived effect of the agency integration was decreased burden for individuals seeking services by having a centralized location for the administrative processes required to access services (Table 1). Interviewees also viewed integration as beneficial in that it increased awareness of dual IDD and mental health diagnoses among important stakeholder groups including both mental health and IDD service providers and advocates and policymakers making decisions affecting services for this population. Interviewees also described increased opportunities for collaborations between IDD and mental health staff following agency integration; however, it was unclear how much of this collaboration at the agency level was also reflected at the service delivery level. Finally, interviewees described increased system complexity as a negative effect of agency integration. Integrating the IDD agency into the mental health department within the larger Health and Human Services Commission added many layers in the structure of the agency, requiring redefinition of roles and responsibilities and longer timelines for processes affecting service delivery. Interviewees indicated that the state was planning to move the IDD agency out of the mental health department and into its own standalone department within the Health and Human Services Commission in part because of this increased complexity related to integrating the two agencies. This move took place in the fall of 2022, after interviews were completed.

**Table 1:** Interviewee perceptions of Texas agency integration effects

Theme	Representative quotes
Decreased administrative burden for those seeking services	“I think the goal was really to do just what we’re talking about, which is try to get all of disability and human services programs into one agency, for coordination of care, continuity of care, make it easier on people receiving services to not have to go to three different agencies, if they’re trying to get services for three distinct needs, kind of thing” “And, you know, bringing that within to streamline services because there were so many families who were seeking intellectual and developmental disability (IDD) services who also needed mental health services”
Increased awareness of co-occurring IDD and mental health diagnoses among providers and policymakers	“I mean, I think, before, we were victims of the whole diagnostic overshadowing conversation, where a person with IDD presents at the local level, and everybody thinks they’re just being challenging or difficult—and really, they’re depressed, or they’re having hallucinations. And really, just the integration raised awareness to that need, and that, in turn, hopefully, helped people gain more access” “I think one of the biggest benefits to that which is kind of like an abstract concept is the awareness that people with IDD can experience mental health conditions. I think a lot, at least, from the legislative side, a lot of advocates weren’t even aware of it or still not aware of it, but when someone goes to talk to [the head of the department] and they see the title and intellectual developmental disability services, I think that really heightens people’s awareness that this is actually an issue because so many advocates—so many mental health advocates have no clue about individuals with intellectual and developmental disabilities. And that these people can also experience mental health” “You know, whether you believe that consolidating all these agencies and creating this mega commission was a good idea or not, I think bringing these two pieces together was certainly a good move towards at least getting people to think about the fact that, yeah, these people do need some services”
Increased opportunities for collaboration	“When you have state leaders over various programs put into one division, you get to know each other, and you learn how to develop collaborations, and then that flows into your service delivery system, right?”
Increased system complexity	“So, all the complexities of [integration] and trying to define and understand what people’s roles and lanes are has a downstream impact to how we deliver services to the clients and if we can’t figure that stuff out of state office, it just creates– yeah, I think it impacts the quality of the work that gets done” “There are so many layers now. We’re not as flat as we used to be and so, getting things approved is harder. So, that means our program staff had to build longer timelines. They had to—it just makes it harder to get our work done because the timelines are much longer because there are many more approval chains than there have been previously” “And I will say the Department of Aging and Disability Services (DADS) system did switch over to the Health and Human Services Commission however, when you go on to state websites or things like that, it still refers to DADS quite a bit and they didn’t put a lot of tools in place for that to be a seamless transition. So, you still do all the state issued forms and you still do all the things that you used to do as it was with DADS, but I feel like it’s a little more complicated because of the systems you have to go through, but then on top of that it’s not real clear on the process for it”

Quotes from semi-structured qualitative interviews with Texas intellectual and developmental disability and mental health state agency and advocacy organization leaders (N = 8) conducted April to July 2022

### Quantitative Results

In the quantitative analysis, state-year level baseline characteristics were similar between treatment and control states with no significant differences between Texas and the comparison states (Table 2). During the baseline period in Texas, a majority of individuals with co-occurring cognitive disabilities and mental health conditions had at least one mental health outpatient visit (52%) or prescription medication (52%) per year; emergency department visits (5%) and inpatient stays (3%) were less common (Appendix B in Supplementary material). Augmented synthetic control weights are provided in Supplementary material Appendix C. As designed, the augmented synthetic control approach achieved good balance on pre-integration outcomes; mean outcomes during the baseline period were identical in Texas and the synthetic control (Appendix D in Supplementary material).

**Table 2:** Unweighted state-year baseline characteristics, 2014–2016

	Texas	Comparison states^a^
Age (mean, standard deviation)	55.9 (3.5)	54.1 (4.2)
Sex (%)		
Female	64.8	61.6
Male	35.2	38.4
Race/ethnicity (%)		
White, non-Hispanic	26.4	51.9
Black, non-Hispanic	25.9	20.7
Asian, non-Hispanic	1.4	0.7
Other, non-Hispanic	4.2	4.7
Hispanic	42.1	22.0
Household annual income		
< $25,000	63.9	71.4
$25,000–$49,999	19.1	15.2
$50,000–$74,999	11.4	6.7
$75,000–$99,999	2.7	3.8
$100,00 +	2.9	2.9
Highest degree attained (%)		
Less than high school	37.8	31.3
High school	54.5	57.2
Bachelor’s degree	2.9	7.8
Graduate degree or higher	4.7	3.8
Employment (%)		
Employed	13.5	16.3
Unemployed	86.5	83.7
Marital status (%)		
Single	22.8	34.2
Married	22.1	21.4
Widowed	21.0	15.9
Divorced	34.2	28.4
Insurance coverage (%)		
Any medicare	54.8	52.1
Any medicaid	41.7	53.3
Any private insurance	20.5	18.8
Full year uninsured	10.2	8.4
Autism spectrum disorder (%)	0.0	0.6
Mood disorder (%)	19.9	17.0
Anxiety disorder (%)	53.4	65.3
Schizophrenia (%)	2.2	1.3

*p < 0.05

^a^Control states are the ten states (AZ, CT, FL, NM, NY, OH, OK, OR, SC, TN) with separate intellectual and developmental disability (IDD) and mental health agencies for the entire study period and sufficient sample size in MEPS data for analysis

**Fig. 2:**
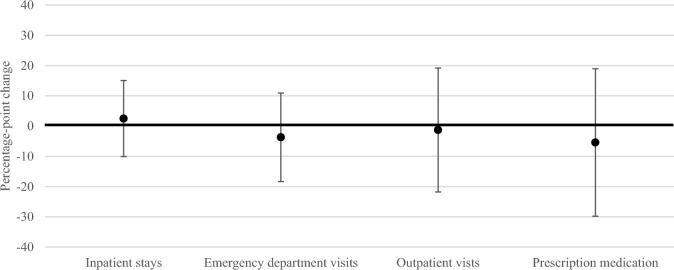
Change in the proportion of individuals receiving any mental health related inpatient, emergency department, outpatient, or prescription medication services, per year, attributable to Texas state agency integration in the first four years of implementation, 2017–2020^a^

Results of augmented synthetic control analyses showed small in magnitude, non-statistically significant changes in mental health service outcomes attributable to agency integration in Texas (Fig. 2). In the first four years following implementation (2017–2020), the proportion of individuals with at least one mental health related inpatient stay per year was slightly increased (ATT: 2.48 percentage points, 95% confidence interval (CI) − 10.10, 15.05) and the proportion of individuals with at least one mental health related emergency department visit (ATT: − 3.70 percentage points, 95% CI − 18.34, 10.94), outpatient visit (ATT: − 1.29 percentage points, 95% CI − 21.79, 19.20), or prescription medication (ATT: − 5.42 percentage points, 95% − 29.80, 18.96) per year were slightly decreased in Texas relative to what would have been expected in the absence of agency integration. Because inpatient stays and emergency department visits were infrequent in this sample, changes in the number of services per year were only estimable for outpatient visit and prescription medication outcomes. We observed no statistically significant change in the mean number of outpatient visits (ATT: − 0.60 visits, 95% CI: − 5.71, 4.51) or the mean number of prescription medications (ATT: 0.10 medications, − 0.49, 0.68) per person, per year attributable to agency integration. These findings held across analyses assessing the change in outcomes for each year since implementation separately (Appendix E in Supplementary material), and with the expanded definitions of cognitive disability and mental health conditions, exclusion of individuals aged 55 and older, implementation date of 2016, and inclusion of different sets of covariates (Appendix F in Supplementary material).

### Triangulation of Qualitative and Quantitative Data

Interviewees described significant changes that took place to integrate the Texas disability and mental health agencies in 2017 and possible mechanisms that could lead to improved access to mental health services for individuals with co-occurring IDD and mental health conditions. These mechanisms included: decreased administrative burden for individuals seeking services, increased awareness of co-occurring IDD and mental health conditions, and increased opportunities for inter-agency collaboration. These perceived benefits, however, were described at the agency level, and may not have been realized in actual service delivery. As summarized by one interviewee,

“Whether you believe that consolidating all these agencies and creating this mega commission was a good idea or not, I think bringing these two pieces together was certainly a good move ... It hasn’t necessarily trickled down to the local level to our safety net community centers, on the ground service delivery. It has in a few places … but there’s a real fire wall between [the IDD and mental health local authorities] … and I think there’s still some structural billing code problems that make it difficult to receive services from both sides. But I think there are also very much some cultural barriers and maybe power or territorial kind of barriers that, ‘this person is mine,’ even though they may not know what to do with them.”

Interviewees also described increased system complexity which had the potential to negatively impact mental health service delivery and individual-level service outcomes. Reflecting these limitations, we did not observe any statistically significant change, either positive or negative, in mental health service use among individuals with both cognitive disability and mental health conditions attributable to agency integration. Taken together, these findings suggest that integrating state disability and mental health agencies alone may be insufficient to result in meaningful service-delivery and patient-level changes in access to mental health services for individuals with co-occurring cognitive disabilities, including IDD specifically, and mental health conditions.

## Discussion

While the need for inter-agency coordination has been raised in the context of improving mental health services for individuals with IDD, (Pinals et al., 2021b) we observed no changes in mental health service outcomes among people with cognitive disabilities and mental health conditions following integration of state disability and mental health agencies in Texas. Prior literature on the role of mental health agencies in evidence-based practice delivery finds that agency involvement, funding, and workforce development are important aspects of improving individual-level outcomes (Ghose et al., 2022; Isett et al., 2008; Jones et al., 2014). In this analysis, interviewees described positive structural changes at the agency level (i.e., changing the name of the agency and bringing staff and leadership together), but with limited—or even complicated—impacts on service delivery. In fact, important service delivery-level barriers including billing and cultural differences persisted post-agency integration. These findings highlight the need to consider multiple levels of intervention in implementing changes to improve mental health services for indiivduals with co-occurring IDD and mental health conditions.

IDD services are typically funded and delivered through Medicaid Home and Community Based Services (HCBS) waiver programs, while public mental health services are funded by state or (non-HCBS) Medicaid funds. This funding is often tied to diagnostic or functional eligibility criteria that focuses on only one part of an individual’s true service need (SAMHSA, 2021; Pinals et al., 2021b). Moving away from these discrete eligibility categories and blending or braiding service funding streams may help to increase access to services across systems. Blending or braiding of funding streams would require changes to policies at the state and/or federal levels. One example is Delaware’s Assertive Community Integration and Support Team that uses state funds to coordinate a full range of services and supports for individuals with both IDD and mental health conditions (SAMHSA, 2021). Recent moves at the federal level to increase the use of the Medicaid Early Periodic Screening, Diagnostic, and Treatment benefit for children is another example that may also help to ensure funding for a range of mental health and disability services through Medicaid without relying on categorical funding criteria (Guth & Williams, 2022).

Even with agency integration and less rigid funding streams, efforts to improve services for individuals with co-occurring cognitive disabilities, and specifically IDD, and mental health conditions will be limited without consideration of those providing services. While stakeholders reported that agency integration increased awareness of dual IDD and mental health diagnoses, most mental health providers do not receive training to treat people with IDD and most IDD providers are not trained to work with individuals with mental health conditions (Ervin et al., 2014; Kramer et al., 2019; Pinals et al., 2021a; Whittle et al., 2018). Individuals with IDD often have different presentations of mental health conditions than the general population, can have challenges communicating their internal states due to language or cognitive impairment, and may require accommodations such as assistive communication technologies (Fletcher et al., 2007). Recent research has shown that in general, providers are unfamiliar with how to accommodate patients with disabilities and are unwilling to work with patients with disabilities, even actively discharging them from their practice (Lagu et al., 2022). Interventions such as specialized training, decision support, or incentives such as enhanced reimbursement for providing services for individuals with dual diagnoses may be warranted.

Findings of this study should be viewed in light of its limitations. For qualitative data collection, interviewees were asked about agency integration that occurred 5 years ago and responses may be subject to recall bias, though the integration is a large and on-going process, so interviewees were likely able to recall key themes. In an attempt to ensure we gathered accurate information about the process, we spoke to individuals who were present at the agencies at the time of agency integration and cross-referenced contemporaneous documents as necessary (e.g., to check dates). Additionally, as our analysis focused on the process and outcomes of agency integration, we focused our interview recruitment on individuals in the state agency and advocacy organizations that interact with these agencies. The perspectives of these leaders may differ from the perspectives of other stakeholder groups including providers and people with co-occurring IDD and mental health conditions using the services. Our quantitative analysis used an augmented synthetic control approach. In settings with limited sample size such as this (N = 11 states), this method can produce high-variance estimates resulting in findings that may be clinically significant despite statistical non-significance (Griffin et al., 2023). In this analysis, though, while the confidence intervals are large, the point estimates indicate relatively small effects. Also in the quantitative analysis, the MEPS data does not include individuals living in institutions (e.g., state supported living centers) and therefore may not be generalizable to individuals with higher service needs who reside in those settings. A significant limitation of the MEPS data is the inability to directly measure IDD. Indeed, in our analytic sample, few people with a diagnosis of autism spectrum disorder, a common developmental disability, were identified. We attempt to mitigate this concern by conducting a sensitivity analysis using an alternate definition of cognitive disability. Additionally, the mean age of the analytic sample is approximately 55. While our focus was on individuals with IDD, the original Texas DADS agency was also tasked with programming for aging populations that may be captured in the cognitive disability population alongside individuals with IDD specifically. In order to better approximate an IDD sample, we conduct a sensitivity analysis limiting the sample to those aged 54 and younger who may be less likely to have been categorized as having a cognitive disability due to an age-related condition. Findings from sensitivity analyses were similar to main results. Finally, the MEPS analysis did not use a longitudinal cohort; however, survey respondent characteristics remained fairly stable over time in both the treatment and control groups. Many limitations of the MEPS data for this analysis, including the relatively small sample size and inexact definition of the target population, point to the need for better data collection on people with disabilities in general and people with IDD specifically, across a range of services and outcomes (Dorfman & Landes, 2022).

## Conclusion

While state agency and advocacy organization leaders identified some promising impacts of state disability and mental health agency integration, we did not find evidence of impacts on mental health service use among individuals with co-occurring cognitive disabilities and mental health conditions. Interviewees described the limited impact of these agency-level structural changes on downstream service delivery. Future efforts to improve mental health services for individuals with co-occurring conditions should consider interventions across multiple levels of the service system.

## Supplementary Information

Below is the link to the electronic supplementary material.

Supplementary file1 (DOCX 204 kb)
