# Supplementary material for: Effects of Texas State Agency Integration on Mental Health Service Use Among Individuals with Co-occurring Cognitive Disabilities and Mental Health Conditions
Source: Community Ment Health J. 2024 Aug 1;61(1):111–21. doi: 10.1007/s10597-024-01332-0 (PMC11703680; doi:10.1007/s10597-024-01332-0)
Supplement: Supplementary file 1 — Supplementary file1 (DOCX 204 kb) [file 10597_2024_1332_MOESM1_ESM.docx]

Effects of Texas state agency integration on mental health service use among individuals with co-occurring cognitive disabilities and mental health conditions

**Appendices**

| **Appendix A** | Qualitative interview guide | pages 2-3 |
| --- | --- | --- |
| **Appendix B** | Trends in mental health service use in Texas and control states, 2014-2020 | pages 4-5 |
| **Appendix C** | Augmented synthetic control weights | page 6 |
| **Appendix D** | Change in mental health service outcomes, per year, attributable to agency integration during its first 4 years of implementation (2017-2020) | pages 7-8 |
| **Appendix E** | Augmented synthetic control time series outcomes | pages 9-14 |
| **Appendix F** | Sensitivity analyses | pages 15-20 |

**Appendix A. Qualitative interview guide**

**A. Introduction**

*Purpose of this study:* We’re talking with individuals who are familiar with mental health services for individuals with co-occurring intellectual/developmental disabilities and mental illness in Texas. We are interested in learning about delivery and quality of mental health services for this population and the role state agencies play in these services. We are particularly interested understanding how the integration of the Department of Aging and Disability Services into Health and Human Services has affected mental health services delivery for this group. We’re interested in hearing your thoughts and opinions about these topics based on your experiences.

*What we will do today:* Our interview today will take approximately 60 minutes. As a reminder, this interview is meant to be like a conversation. Your participation is voluntary, and you can stop the interview at any time. If you would prefer not to answer a question, please just say so. With your permission, I’m going to record the interview. The interview will be transcribed, and all answers will be anonymized. Do I have your permission to record this interview? Do you have any questions for me before we start? I am going to start recording now.

**B. Overview**

- To start off, can you give me a brief overview of your role as it relates to mental health services for individuals with co-occurring IDD and mental illness in [your state]?
- In your opinion, what is the current state of mental health services for this population in [your state]?
  - Does this vary across populations (e.g., children vs. adults, Medicaid insured vs. privately insured, autism spectrum disorder (ASD) vs. other diagnoses, etc.)?

**C. Current role of agencies in mental health services**

- What role do the state agencies currently play in the following domains for people with co-occurring IDD and mental illness: [For each domain, do these roles vary across populations (e.g., children vs. adults, Medicaid insured vs. privately insured, ASD vs. other diagnoses, etc.)?]
  - Providing mental health services directly?
  - Coordinating mental health services?
  - Regulating mental health services?
- Are there other roles beyond these that state agencies play in mental health services for those with IDD? If yes, what are these roles? Do they vary across populations (if yes, how)?

**D. Barriers and facilitators to mental health services**

- What do you see as the primary barrier(s) to state agencies effectively meeting the mental health service needs of individuals with IDD and mental illness?
  - Prompts: administrative barriers, funding, personnel, etc.
  - Does this vary across populations (e.g., children vs. adults, Medicaid insured vs. privately insured, ASD vs. other diagnoses, etc.)?
- What do you see as the primary facilitator(s) to state agencies effectively meeting the mental health service needs of individuals with IDD and mental illness?
  - Prompts: administrative barriers, funding, personnel, etc.
  - Does this vary across populations (e.g., children vs. adults, Medicaid insured vs. privately insured, ASD vs. other diagnoses, etc.)?

**E. Agency restructuring**

- Were you present for the restructuring when the Department of Aging and Disability Services moved into the Department of Health and Human Services? [If yes, continue; if no, skip to conclusion]
- What was your role during this process?

**E.1. Restructuring process (Planning/Engaging/Executing)**

- In your view, what was the goal for restructuring the agencies?
- Specific goals related to individuals with IDD and mental illness?
- Can you talk me through the timeline of the restructuring?
- From the perspective of your role/agency, who and what was involved in the process of restructuring?
- From your perspective was there anything that made this process easier? More difficult?

**E.2. Restructuring outcomes (Reflecting and evaluating)**

- Has the restructuring changed the day-to-day agency work in the following domains for people with co-occurring IDD and mental illness: [For each domain, do these roles vary across populations (e.g., children vs. adults, Medicaid insured vs. privately insured, ASD vs. other diagnoses, etc.)?]
  - Providing mental health services directly?
  - Coordinating mental health services?
  - Regulating mental health services?
- Are there other changes beyond these domains to the work of state agencies related to mental health services for those with IDD and mental illness?
- In your opinion have there been specific **benefits** – either initially or longer-term – to this restructuring as related to mental health services for people with IDD and mental illness?
  - Do these benefits or draw backs vary across populations (e.g., children vs. adults, Medicaid insured vs. privately insured, ASD vs. other diagnoses, etc.)?
- In your opinion have there been specific **drawbacks** – either initially or longer-term – to this restructuring as related to mental health services for people with IDD and mental illness?
  - Do these benefits or draw backs vary across populations (e.g., children vs. adults, Medicaid insured vs. privately insured, ASD vs. other diagnoses, etc.)?
- In your opinion have there been specific **unintended consequences** – either initially or longer-term – to this restructuring as related to mental health services for people with IDD and mental illness?
  - Do these benefits or draw backs vary across populations (e.g., children vs. adults, Medicaid insured vs. privately insured, ASD vs. other diagnoses, etc.)?
- Do you feel like the goals of the restructuring are being met? Why or why not?
  - Specific goals related to individuals with IDD and mental illness?
- Do you think restructuring has influenced **access** to mental health services for individuals with co-occurring IDD and mental illness? Why or why not?
  - Does this differ for different subgroups of people (e.g., children vs. adults, Medicaid insured vs. privately insured, ASD vs. other diagnoses, etc.)?
- Do you think restructuring has influenced **quality** of mental health services for individuals with co-occurring IDD and mental illness? Why or why not?
  - Does this differ for different subgroups of people (e.g., children vs. adults, Medicaid insured vs. privately insured, ASD vs. other diagnoses, etc.)?

**F. Conclusion**

- Is there anything else you think it would be important for us to know or think about as we move forward with our project?
- Is there anyone else you think we should contact for an interview?
- Would you be willing to participate in a brief (~15 minute) follow up interview in the future to discuss preliminary findings from this study?

Thank you very much for your time.

**Appendix B. Trends in mental health service use in Texas and control states, 2014-2020**

**Appendix C. Augmented synthetic control weights**

**Appendix D. Change in mental health service outcomes, per year, attributable to agency integration during its first 4 years of implementation (2017-2020)**

Change in proportion of individuals receiving any mental health service, per year, attributable to agency integration during its first 4 years of implementation

|  | | Mean proportion of individuals receiving any mental health service, per year | | Difference (percentage points) | Difference-in-differences estimate of change attributable to integration (percentage points) ^1^ |
| --- | --- | --- | --- | --- | --- |
|  |  | 3 years pre | 4 years post |  |  |
| Inpatient stays | |  |  |  |  |
|  | Texas | 2.61 | 2.87 | 0.26 | 2.48 (-10.10, 15.05) |
|  | Synthetic control | 2.61 | 0.39 | -2.22 |  |
| Emergency department visits | |  |  |  |  |
|  | Texas | 5.12 | 2.48 | -2.64 | -3.70 (-18.34, 10.94) |
|  | Synthetic control | 5.12 | 6.18 | 1.06 |  |
| Outpatient visits | |  |  |  |  |
|  | Texas | 52.27 | 51.33 | -0.94 | -1.29 (-21.79, 19.20) |
|  | Synthetic control | 52.27 | 52.62 | 0.35 |  |
| Prescription medications | |  |  |  |  |
|  | Texas | 52.25 | 58.97 | 6.72 | -5.42 (-29.80, 18.96) |
|  | Synthetic control | 52.25 | 64.38 | 12.13 |  |

^1^Estimates may differ from differences because of rounding

Augmented synthetic control analyses were used to estimate the changes in outcomes between the pre-integration and post-integration periods in Texas and the synthetic control group. The results in this figure show the percentage-point difference in the proportion of patients with any mental health related service use, per year, in the 3 years before versus 4 years after agency integration in Texas versus the synthetic control group. Analyses were adjusted for state fixed effects and state-year aggregated measures of age, race/ethnicity, household income, education level, employment status, marital status, insurance coverage, autism spectrum disorder diagnosis, and mental health diagnosis (mood disorder, anxiety disorder, schizophrenia).

Change in number of mental health services received among individuals receiving at least one service, per year, attributable to agency integration during its first 4 years of implementation

|  | | Mean number of mental health services received per person, per year, among individuals with ≥ 1 service | | Difference (number of services) | Difference-in-differences estimate of change attributable to integration (number of services) ^1^ |
| --- | --- | --- | --- | --- | --- |
|  |  | 3 years pre | 4 years post |  |  |
| Outpatient visits | |  |  |  |  |
|  | Texas | 4.56 | 6.14 | 1.58 | -0.60 (-5.71, 4.51) |
|  | Synthetic control | 4.56 | 6.74 | 2.18 |  |
| Prescription medications | |  |  |  |  |
|  | Texas | 1.51 | 1.58 | 0.07 | 0.10 (-0.49, 0.68) |
|  | Synthetic control | 1.51 | 1.49 | -0.02 |  |

^1^Estimates may differ from differences because of rounding

Augmented synthetic control analyses were used to estimate the changes in outcomes between the pre-integration and post-integration periods in Texas and the synthetic control group. The results in this table show the difference in the number of mental health services used, per year, among individuals with at least one service use in each year in the 3 years before versus 4 years after agency integration in Texas versus the synthetic control group. Analyses were adjusted for state fixed effects and state-year aggregated measures of age, race/ethnicity, household income, education level, employment status, marital status, insurance coverage, autism spectrum disorder diagnosis, and mental health diagnosis (mood disorder, anxiety disorder, schizophrenia).

**Appendix E. Augmented synthetic control time series outcomes**

Change in proportion of individuals receiving any mental health **inpatient stay**, per year, attributable to agency integration

|  | Change in the mean proportion of patients receiving any mental health services, per year, attributable to integration (percentage points) (95% CI) |
| --- | --- |
| 2017 | 0.6 (-7.2, 8.3) |
| 2018 | 0.6 (-7.1, 8.4) |
| 2019 | 1.1 (-6.7, 8.8) |
| 2020 | 7.6 (-0.1, 15.4) |

Augmented synthetic control analyses were used to estimate the changes in outcomes between the pre-integration and post-integration periods in Texas and the synthetic control group. The results in this table show the percentage-point difference in the proportion of patients with any mental health related service use in each year following agency integration in Texas versus the synthetic control group. Analyses were adjusted for state fixed effects and state-year aggregated measures of age, race/ethnicity, household income, education level, employment status, marital status, insurance coverage, autism spectrum disorder diagnosis, and mental health diagnosis (mood disorder, anxiety disorder, schizophrenia).


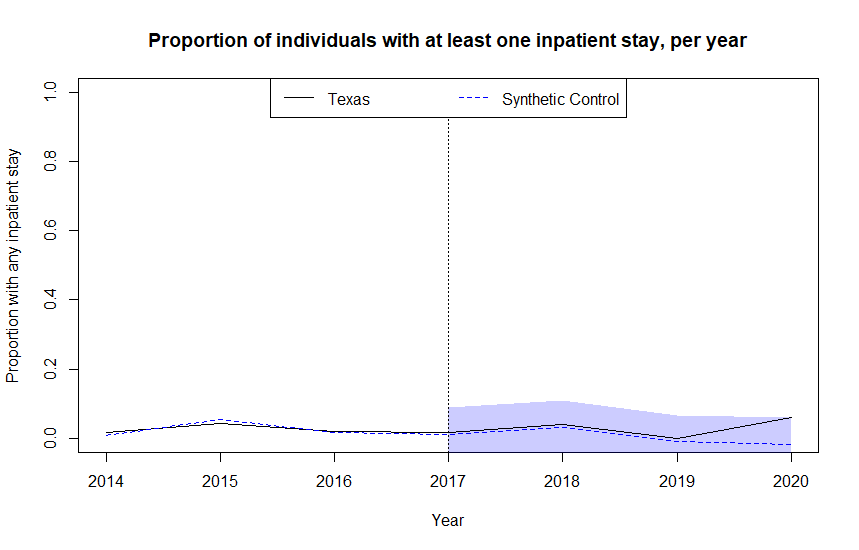


Change in proportion of individuals receiving any mental health **emergency department visit**, per year, attributable to agency integration

|  | Change in the mean proportion of patients receiving any mental health services, per year, attributable to integration (percentage points) (95% CI) |
| --- | --- |
| 2017 | -3.5 (-12.5, 5.5) |
| 2018 | -7.9 (-16.9, 1.2) |
| 2019 | -0.9 (-9.9, 8.1) |
| 2020 | -2.5 (-11.5, 6.5) |

Augmented synthetic control analyses were used to estimate the changes in outcomes between the pre-integration and post-integration periods in Texas and the synthetic control group. The results in this table show the percentage-point difference in the proportion of patients with any mental health related service use in each year following agency integration in Texas versus the synthetic control group. Analyses were adjusted for state fixed effects and state-year aggregated measures of age, race/ethnicity, household income, education level, employment status, marital status, insurance coverage, autism spectrum disorder diagnosis, and mental health diagnosis (mood disorder, anxiety disorder, schizophrenia).


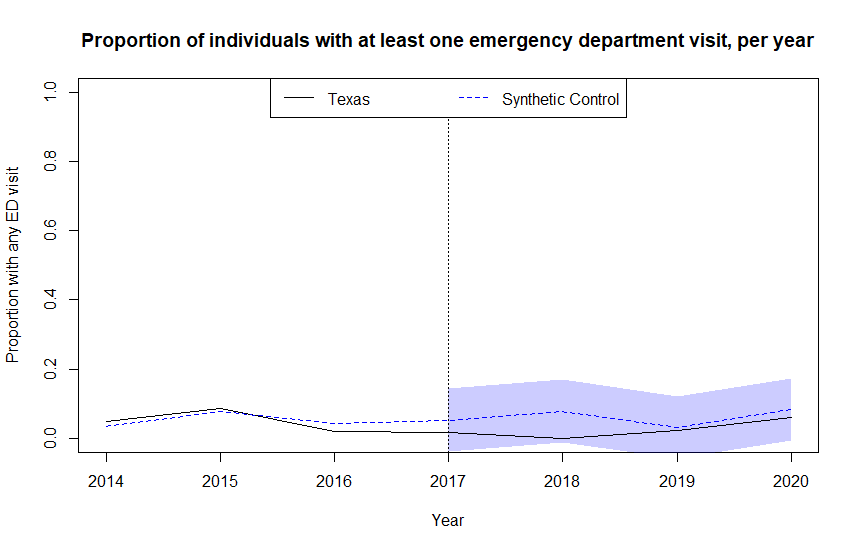


Change in proportion of individuals receiving any mental health **outpatient visit**, per year, attributable to agency integration

|  | Change in the mean proportion of patients receiving any mental health services, per year, attributable to integration (percentage points) (95% CI) |
| --- | --- |
| 2017 | -9.9 (-22.6, 2.7) |
| 2018 | -0.6 (-13.2, 12.1) |
| 2019 | -2.2 (-14.8, 10.5) |
| 2020 | 7.5 (-5.2, 20.1) |

Augmented synthetic control analyses were used to estimate the changes in outcomes between the pre-integration and post-integration periods in Texas and the synthetic control group. The results in this table show the percentage-point difference in the proportion of patients with any mental health related service use in each year following agency integration in Texas versus the synthetic control group. Analyses were adjusted for state fixed effects and state-year aggregated measures of age, race/ethnicity, household income, education level, employment status, marital status, insurance coverage, autism spectrum disorder diagnosis, and mental health diagnosis (mood disorder, anxiety disorder, schizophrenia).


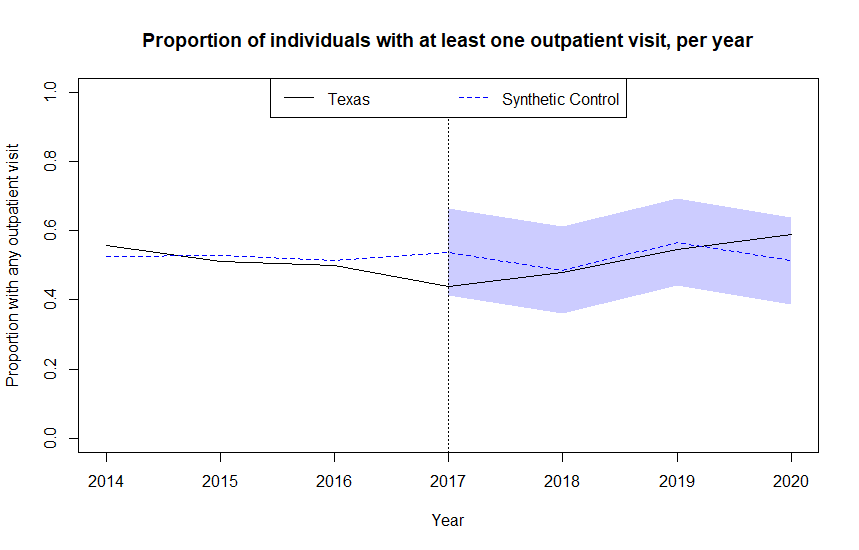


Change in mean proportion of individuals receiving any mental health **prescription medication**, per year, attributable to agency integration

|  | Change in the mean proportion of patients receiving any mental health services, per year, attributable to integration (percentage points) (95% CI) |
| --- | --- |
| 2017 | -10.1 (-25.1, 4.9) |
| 2018 | -4.2 (-19.2, 10.8) |
| 2019 | -9.9 (-24.9, 5.1) |
| 2020 | 2.6 (-12.4, 17.6) |

Augmented synthetic control analyses were used to estimate the changes in outcomes between the pre-integration and post-integration periods in Texas and the synthetic control group. The results in this table show the percentage-point difference in the proportion of patients with any mental health related service use in each year following agency integration in Texas versus the synthetic control group. Analyses were adjusted for state fixed effects and state-year aggregated measures of age, race/ethnicity, household income, education level, employment status, marital status, insurance coverage, autism spectrum disorder diagnosis, and mental health diagnosis (mood disorder, anxiety disorder, schizophrenia).


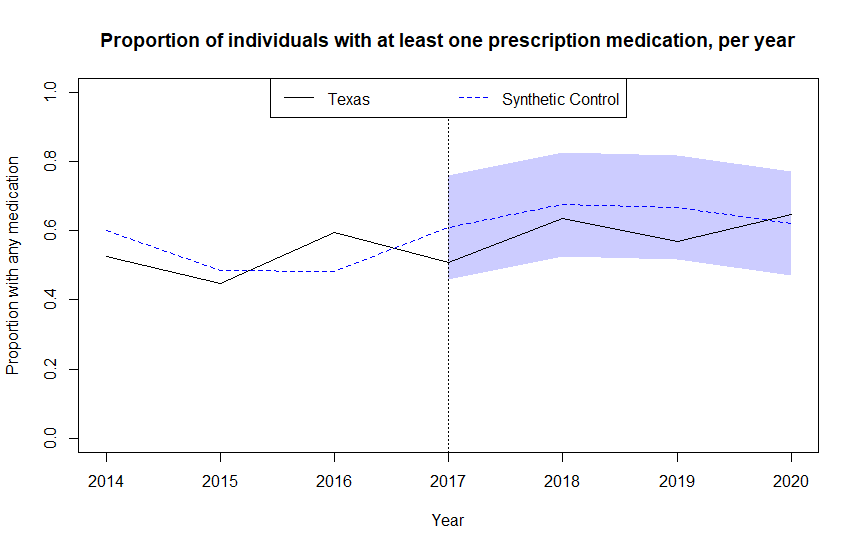


Change in number of mental health **outpatient visits** per person, per year, among individuals with ≥ 1 service attributable to agency integration

|  | Change in the mean number of mental health services received per person, per year, among individuals with ≥ 1 service attributable to integration (number of services) (95% CI) |
| --- | --- |
| 2017 | -0.5 (-3.7, 2.6) |
| 2018 | -2.0 (-5.2, 1.1) |
| 2019 | -1.6 (-4.7, 1.6) |
| 2020 | 1.7 (-1.4, 4.9) |

Augmented synthetic control analyses were used to estimate the changes in outcomes between the pre-integration and post-integration periods in Texas and the synthetic control group. The results in this table show the difference in the number of mental health services used, per year, among individuals with at least one service use in each year following agency integration in Texas versus the synthetic control group. Analyses were adjusted for state fixed effects and state-year aggregated measures of age, race/ethnicity, household income, education level, employment status, marital status, insurance coverage, autism spectrum disorder diagnosis, and mental health diagnosis (mood disorder, anxiety disorder, schizophrenia).


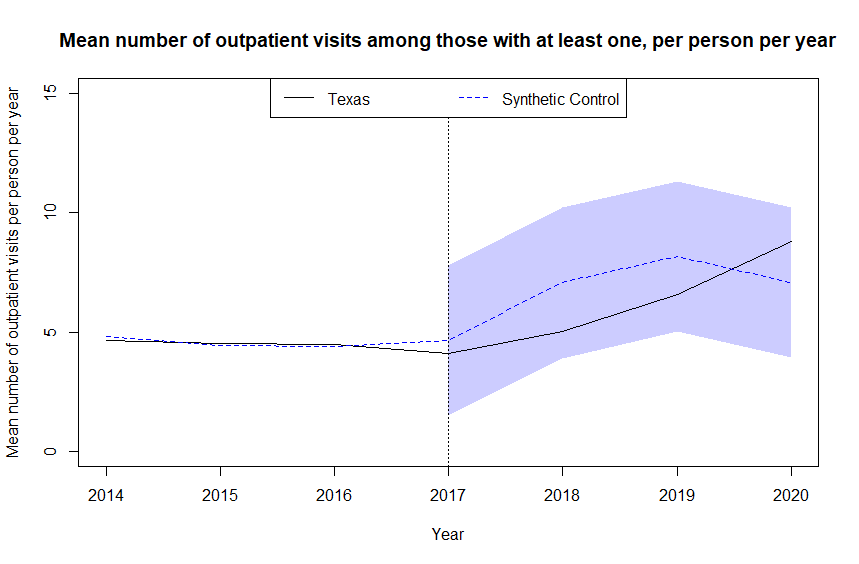


Change in number of mental health **prescription medications** per person, per year, among individuals with ≥ 1 service attributable to agency integration

|  | Change in the mean number of mental health services received per person, per year, among individuals with ≥ 1 service attributable to integration (number of services) (95% CI) |
| --- | --- |
| 2017 | -0.1 (-0.4, 0.3) |
| 2018 | 0.02 (-0.3, 0.4) |
| 2019 | 0.01 (-0.3, 0.5) |
| 2020 | 0.3 (-0.02, 0.7) |

Augmented synthetic control analyses were used to estimate the changes in outcomes between the pre-integration and post-integration periods in Texas and the synthetic control group. The results in this table show the difference in the number of mental health services used, per year, among individuals with at least one service use in each year following agency integration in Texas versus the synthetic control group. Analyses were adjusted for state fixed effects and state-year aggregated measures of age, race/ethnicity, household income, education level, employment status, marital status, insurance coverage, autism spectrum disorder diagnosis, and mental health diagnosis (mood disorder, anxiety disorder, schizophrenia).


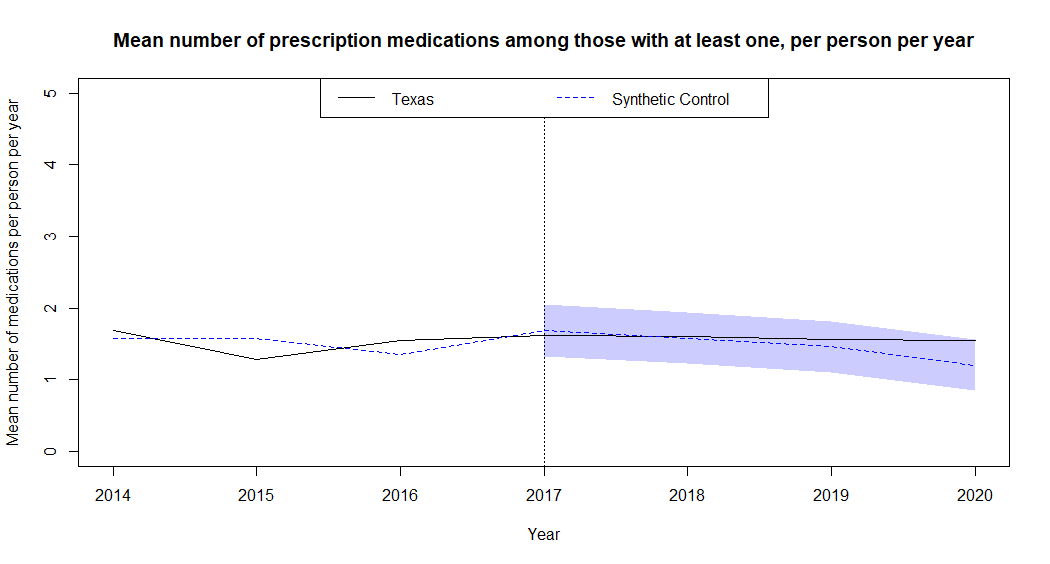


**Appendix F. Sensitivity analyses**

Analyses with an alternative definition of cognitive disability

Cognitive disability defined as: cognitive limitation (experienced confusion or memory loss, had problems making decisions, or required supervision for their own safety) OR serious cognitive difficulty (difficulty concentrating, remembering, or making decisions)

|  | Change in the mean proportion of patients receiving any mental health services, per year, attributable to integration (percentage points) (95% CI) |
| --- | --- |
| Inpatient stays | 1.63 (-4.40, 7.65) |
| Emergency department visits | 2.83 (-7.84, 13.50) |
| Outpatient visits | 6.32 (-26.35, 38.99) |
| Prescription medications | 3.08 (-33.84, 40.00) |

Augmented synthetic control analyses were used to estimate the changes in outcomes between the pre-integration and post-integration periods in Texas and the synthetic control group. The results in this table show the percentage-point difference in the proportion of patients with any mental health related service use, per year, in the 3 years before versus 4 years after agency integration in Texas versus the synthetic control group. Analyses were adjusted for state fixed effects and state-year aggregated measures of age, race/ethnicity, household income, education level, employment status, marital status, insurance coverage, autism spectrum disorder diagnosis, and mental health diagnosis (mood disorder, anxiety disorder, schizophrenia).

|  | Change in the mean number of mental health services received per person, per year, among individuals with ≥ 1 service attributable to integration (number of services) (95% CI) |
| --- | --- |
| Outpatient visits | -2.11 (-9.56, 5.35) |
| Prescription medications | 0.04 (-0.36, 0.44) |

Augmented synthetic control analyses were used to estimate the changes in outcomes between the pre-integration and post-integration periods in Texas and the synthetic control group. The results in this table show the difference in the number of mental health services used, per year, among individuals with at least one service use in the 3 years before versus 4 years after agency integration in Texas versus the synthetic control group. Analyses were adjusted for state fixed effects and state-year aggregated measures of age, race/ethnicity, household income, education level, employment status, marital status, insurance coverage, autism spectrum disorder diagnosis, and mental health diagnosis (mood disorder, anxiety disorder, schizophrenia).

Analyses with an alternative definition of mental health conditions

Mental health conditions defined as: relevant diagnosis code OR a Patient Health Questionnaire (PHQ) score greater than 2 OR a Kessler Psychological Distress Scale (K6) score greater than 12

|  | Change in the mean proportion of patients receiving any mental health services, per year, attributable to integration (percentage points) (95% CI) |
| --- | --- |
| Inpatient stays | 2.01 (-6.22, 10.23) |
| Emergency department visits | -0.23 (-7.48, 7.02) |
| Outpatient visits | -0.66 (-18.74, 17.42) |
| Prescription medications | 2.78 (-15.46, 21.03) |

Augmented synthetic control analyses were used to estimate the changes in outcomes between the pre-integration and post-integration periods in Texas and the synthetic control group. The results in this table show the percentage-point difference in the proportion of patients with any mental health related service use, per year, in the 3 years before versus 4 years after agency integration in Texas versus the synthetic control group. Analyses were adjusted for state fixed effects and state-year aggregated measures of age, race/ethnicity, household income, education level, employment status, marital status, insurance coverage, autism spectrum disorder diagnosis, and mental health diagnosis (mood disorder, anxiety disorder, schizophrenia).

|  | Change in the mean number of mental health services received per person, per year, among individuals with ≥ 1 service attributable to integration (number of services) (95% CI) |
| --- | --- |
| Outpatient visits | -0.72 (-4.51, 3.07) |
| Prescription medications | -0.86 (-34.29, 32.56) |

Augmented synthetic control analyses were used to estimate the changes in outcomes between the pre-integration and post-integration periods in Texas and the synthetic control group. The results in this table show the difference in the number of mental health services used, per year, among individuals with at least one service use in the 3 years before versus 4 years after agency integration in Texas versus the synthetic control group. Analyses were adjusted for state fixed effects and state-year aggregated measures of age, race/ethnicity, household income, education level, employment status, marital status, insurance coverage, autism spectrum disorder diagnosis, and mental health diagnosis (mood disorder, anxiety disorder, schizophrenia).

Analyses excluding people aged 55 and older

Unweighted state-year baseline characteristics, 2014-2016

|  | | **Texas** | **Comparison states^a^** |
| --- | --- | --- | --- |
| Age (mean, standard deviation) | | 41.0 (4.0) | 41.0 (3.3) |
| Sex (%) | |  |  |
|  | Female | 68.3 | 64.8 |
|  | Male | 31.7 | 35.2 |
| Race/ethnicity (%) | |  |  |
|  | White, non-Hispanic | 25.6 | 51.3* |
|  | Black, non-Hispanic | 33.3 | 24.3 |
|  | Asian, non-Hispanic | 2.0 | 0.9 |
|  | Other, non-Hispanic | 2.5 | 4.6 |
|  | Hispanic | 36.7 | 18.8 |
| Household annual income | |  |  |
|  | < $25,000 | 57.9 | 71.2 |
|  | $25,000-$49,999 | 15.2 | 17.4 |
|  | $50,000-$74,999 | 14.1 | 7.3 |
|  | $75,000-$99,999 | 7.1 | 2.6 |
|  | $100,000+ | 5.7 | 1.5 |
| Highest degree attained (%) | |  |  |
|  | Less than high school | 27.4 | 26.2 |
|  | High school | 66.0 | 57.7 |
|  | Bachelor’s degree | 2.5 | 13.9 |
|  | Graduate degree or higher | 4.2 | 2.2 |
| Employment (%) | |  |  |
|  | Employed | 25.3 | 26.6 |
|  | Unemployed | 74.7 | 73.4 |
| Marital status (%) | |  |  |
|  | Single | 43.7 | 42.5 |
|  | Married | 21.6 | 19.3 |
|  | Widowed | 2.8 | 2.6 |
|  | Divorced | 31.9 | 35.7 |
| Insurance coverage (%) | |  |  |
|  | Any Medicare | 25.6 | 35.6 |
|  | Any Medicaid | 44.1 | 62.2 |
|  | Any private insurance | 25.1 | 20.6 |
|  | Full year uninsured | 19.2 | 9.5 |
| Autism spectrum disorder (%) | | 0.0 | 0.0 |
| Mood disorder (%) | | 30.4 | 22.6 |
| Anxiety disorder (%) | | 50.1 | 64.7 |
| Schizophrenia (%) | | 4.9 | 3.2 |

* p<0.05

^a^ Control states are the ten states (AZ, CT, FL, NM, NY, OH, OK, OR, SC, TN) with separate intellectual and developmental disability (IDD) and mental health agencies for the entire study period and sufficient sample size in MEPS data for analysis.

|  | Change in the mean proportion of patients receiving any mental health services, per year, attributable to integration (percentage points) (95% CI) |
| --- | --- |
| Inpatient stays | 2.18 (-11.51, 15.87) |
| Emergency department visits | -2.07 (-15.48, 11.34) |
| Outpatient visits | -0.88 (-35.07, 33.32) |
| Prescription medications | -6.07 (-52.21, 40.06) |

Augmented synthetic control analyses were used to estimate the changes in outcomes between the pre-integration and post-integration periods in Texas and the synthetic control group. The results in this table show the percentage-point difference in the proportion of patients with any mental health related service use, per year, in the 3 years before versus 4 years after agency integration in Texas versus the synthetic control group. Analyses were adjusted for state fixed effects and state-year aggregated measures of age, race/ethnicity, household income, education level, employment status, marital status, insurance coverage, autism spectrum disorder diagnosis, and mental health diagnosis (mood disorder, anxiety disorder, schizophrenia).

|  | Change in the mean number of mental health services received per person, per year, among individuals with ≥ 1 service attributable to integration (number of services) (95% CI) |
| --- | --- |
| Outpatient visits | -0.04 (-1.07, 0.98) |
| Prescription medications | -0.18 (-1.17, 0.80) |

Augmented synthetic control analyses were used to estimate the changes in outcomes between the pre-integration and post-integration periods in Texas and the synthetic control group. The results in this table show the difference in the number of mental health services used, per year, among individuals with at least one service use in the 3 years before versus 4 years after agency integration in Texas versus the synthetic control group. Analyses were adjusted for state fixed effects and state-year aggregated measures of age, race/ethnicity, household income, education level, employment status, marital status, insurance coverage, autism spectrum disorder diagnosis, and mental health diagnosis (mood disorder, anxiety disorder, schizophrenia).

Analyses with a 2016 implementation date

|  | Change in the mean proportion of patients receiving any mental health services, per year, attributable to integration (percentage points) (95% CI) |
| --- | --- |
| Inpatient stays | 2.57 (-8.09, 13.23) |
| Emergency department visits | -4.17 (-17.79, 9.46) |
| Outpatient visits | 0.03 (-19.16, 19.23) |
| Prescription medications | 6.32 (-27.50, 40.15) |

Augmented synthetic control analyses were used to estimate the changes in outcomes between the pre-integration and post-integration periods in Texas and the synthetic control group. The results in this table show the percentage-point difference in the proportion of patients with any mental health related service use, per year, in the 2 years before versus 5 years after agency integration in Texas versus the synthetic control group. Analyses were adjusted for state fixed effects and state-year aggregated measures of age, race/ethnicity, household income, education level, employment status, marital status, insurance coverage, autism spectrum disorder diagnosis, and mental health diagnosis (mood disorder, anxiety disorder, schizophrenia).

|  | Change in the mean number of mental health services received per person, per year, among individuals with ≥ 1 service attributable to integration (number of services) (95% CI) |
| --- | --- |
| Outpatient visits | -0.78 (-4.62, 3.06) |
| Prescription medications | NA – model inestimable |

Augmented synthetic control analyses were used to estimate the changes in outcomes between the pre-integration and post-integration periods in Texas and the synthetic control group. The results in this table show the difference in the number of mental health services used, per year, among individuals with at least one service use in the 2 years before versus 5 years after agency integration in Texas versus the synthetic control group. Analyses were adjusted for state fixed effects and state-year aggregated measures of age, race/ethnicity, household income, education level, employment status, marital status, insurance coverage, autism spectrum disorder diagnosis, and mental health diagnosis (mood disorder, anxiety disorder, schizophrenia).

Analyses adjusting only for state fixed effects

|  | Change in the mean proportion of patients receiving any mental health services, per year, attributable to integration (percentage points) (95% CI) |
| --- | --- |
| Inpatient stays | 2.69 (-9.85, 15.23) |
| Emergency department visits | -0.26 (-11.60, 11.07) |
| Outpatient visits | -0.10 (-13.71, 13.54) |
| Prescription medications | -12.82 (-91.57, 65.92) |

Augmented synthetic control analyses were used to estimate the changes in outcomes between the pre-integration and post-integration periods in Texas and the synthetic control group. The results in this table show the percentage-point difference in the proportion of patients with any mental health related service use, per year, in the 3 years before versus 4 years after agency integration in Texas versus the synthetic control group.

|  | Change in the mean number of mental health services received per person, per year, among individuals with ≥ 1 service attributable to integration (number of services) (95% CI) |
| --- | --- |
| Outpatient visits | -1.71 (-8.01, 4.58) |
| Prescription medications | -0.12 (-0.59, 0.35) |

Augmented synthetic control analyses were used to estimate the changes in outcomes between the pre-integration and post-integration periods in Texas and the synthetic control group. The results in this table show the difference in the number of mental health services used, per year, among individuals with at least one service use in the 3 years before versus 4 years after agency integration in Texas versus the synthetic control group.

Analyses adjusting for state fixed effects, age, insurance coverage, autism spectrum disorder diagnosis, and mental health diagnosis (mood disorder, anxiety disorder, schizophrenia)

|  | Change in the mean proportion of patients receiving any mental health services, per year, attributable to integration (percentage points) (95% CI) |
| --- | --- |
| Inpatient stays | 1.67 (-9.51, 12.85) |
| Emergency department visits | -3.56 (-17.19, 10.08) |
| Outpatient visits | -4.11 (-24.70, 16.48) |
| Prescription medications | -11.84 (-63.34, 39.66) |

Augmented synthetic control analyses were used to estimate the changes in outcomes between the pre-integration and post-integration periods in Texas and the synthetic control group. The results in this table show the percentage-point difference in the proportion of patients with any mental health related service use, per year, in the 3 years before versus 4 years after agency integration in Texas versus the synthetic control group.

|  | Change in the mean number of mental health services received per person, per year, among individuals with ≥ 1 service attributable to integration (number of services) (95% CI) |
| --- | --- |
| Outpatient visits | -0.01 (-5.47, 5.46) |
| Prescription medications | 0.10 (-0.42, 0.62) |

Augmented synthetic control analyses were used to estimate the changes in outcomes between the pre-integration and post-integration periods in Texas and the synthetic control group. The results in this table show the difference in the number of mental health services used, per year, among individuals with at least one service use in the 3 years before versus 4 years after agency integration in Texas versus the synthetic control group.
